# Editorial: Cellular and molecular mechanisms in metabolic disorders: role of inflammation and oxidative stress

**DOI:** 10.3389/fphar.2025.1580553

**Published:** 2025-03-06

**Authors:** Munkhtuya Tumurkhuu, Shahnawaz Ali Bhat, Md Zakir Hossain, Mohammad Shafiq, Md Saquib Hasnain, Amit Kumar Nayak, Syed Anees Ahmed

**Affiliations:** ^1^ Department of Internal Medicine, School of Medicine, Wake Forest University, Winston-Salem, NC, United States; ^2^ Department of Zoology, Faculty of Life Sciences, Aligarh Muslim University, Aligarh, India; ^3^ Department of Biological Sciences, Aliah University, Kolkata, India; ^4^ Department of Pediatrics, Ann & Robert H. Lurie Children’s Hospital, Northwestern University, Chicago, IL, United States; ^5^ Department of Pharmacy, Marwadi University, Rajkot, India; ^6^ School of Pharmaceutical Sciences, Siksha ‘O’ Anusandhan (Deemed to be University), Bhubaneswar, India; ^7^ Department of Pharmacology and Toxicology, Brody School of Medicine, East Carolina University, Greenville, NC, United States

**Keywords:** metabolic disorder, inflammation, oxidative stress, diabetes, cardiovascular, lifestyle disorder

Metabolic disorders, including diabetes, obesity, and chronic kidney disease (CKD), remain leading contributors to global morbidity and mortality. These conditions are intricately associated with oxidative stress and inflammation, which not only drive their pathophysiology but also exacerbate complications. Oxidative stress results from an imbalance between the production of reactive oxygen species (ROS) and the body’s capacity to detoxify them, while inflammation involves a chronic immune response that contributes to tissue damage and metabolic dysfunction. The articles highlighted in this editorial present a comprehensive overview of recent advancements in elucidating the underlying mechanisms, identifying potential biomarkers, and developing therapeutic interventions that target oxidative stress and inflammation in metabolic disorders.

## Key mechanisms: the role of oxidative stress and inflammation

The interplay between oxidative stress and inflammation plays a crucial role in the pathogenesis of metabolic disorders. At the cellular level, ROS are not merely byproducts of metabolism but also function as potent signaling molecules capable of inducing oxidative damage to proteins, lipids, and DNA. Songtao et al. investigated the role of claudin-2 (CLDN2), a tight junction protein, as a novel biomarker for prediabetes, highlighting its potential for early diagnosis. Their findings indicate that elevated CLDN2 levels are associated with impaired glucose tolerance and inflammation, providing critical insights into prediabetic conditions.

In diabetic retinopathy (DR), Peng et al. highlighted the protective role of DJ-1, a multifunctional protein, in mitigating retinal ganglion cell damage under high-glucose conditions. Their study demonstrated that DJ-1 preserves mitochondrial function and attenuates ROS generation, emphasizing the critical role of mitochondrial homeostasis in diabetic complications. These findings elucidate the molecular pathways linking oxidative stress to tissue damage and pave the way for targeted therapies aimed at preserving cellular integrity.

## Innovative biomarkers and diagnostic tools

Biomarkers play a crucial role in the identification, monitoring, and management of metabolic disorders. In this context, Songtao et al. identified CLDN2 as a potent biomarker and a valuable tool for early detection of prediabetes. This is particularly significant given that prediabetes often progresses asymptomatically and can lead to serious complications if left untreated. Integrating biomarkers like CLDN2 into routine screening protocols could facilitate timely interventions, potentially reversing or delaying disease progression.

Another promising diagnostic strategy involves the oxidative balance score (OBS), which quantifies the balance between dietary and lifestyle-derived antioxidants and pro-oxidants. Wen et al. demonstrated a significant inverse relationship between OBS and CKD risk using NHANES data, highlighting that higher antioxidant intake confers protection against CKD progression. Similarly, Yang et al. established a correlation between higher OBS and reduced serum uric acid levels, underscoring its potential role in hyperuricemia management. These findings emphasize the need for holistic diagnostic tools that integrate oxidative stress dynamics, providing a more comprehensive approach to assessing and mitigating metabolic disorders.

## β-blockers: a new frontier in metabolic modulation

The review by Drygała et al. presents compelling evidence on the metabolic benefits of β-blockers, particularly third-generation agents such as nebivolol and carvedilol. While traditionally employed in the management of cardiovascular disease (CVD), these agents exhibit anti-inflammatory and antioxidant properties that extend beyond their primary pharmacological roles. Nebivolol enhances nitric oxide (NO) bioavailability, thereby reducing oxidative stress and improving endothelial function. Meanwhile, carvedilol modulates inflammatory pathways via the Nrf2/ARE signaling cascade, contributing to cellular protection against oxidative damage.

Beyond their cardiovascular benefits, these agents also demonstrate potential in modulating insulin resistance and glucose metabolism. Nebivolol, owing to its vasodilatory properties, enhances skeletal muscle perfusion, thereby promoting glucose uptake. Similarly, carvedilol has been shown to exert favorable effects on insulin sensitivity and lipid profiles, making these β-blockers particularly beneficial for individuals with metabolic syndrome or type 2 diabetes.

## Therapeutic advancements: targeting oxidative stress and inflammation

Targeting oxidative stress and inflammation has become a fundamental strategy in the treatment of metabolic disorders. Diphenyl diselenide (DPDS), investigated by Wang et al., exemplifies a promising therapeutic agent capable of modulating oxidative stress and gut microbiota in diabetic kidney disease (DKD). The study demonstrated that DPDS improves renal pathology by reducing ROS levels, enhancing antioxidant enzyme activity, and restoring gut microbial balance. Furthermore, the findings underscored the role of the Nrf2/Keap1 signaling pathway in mediating the protective effects of DPDS, reinforcing its potential as a therapeutic intervention for oxidative stress-related renal complications.

Nutritional interventions represent another promising avenue for mitigating oxidative stress and inflammation. Hu et al. investigated *Vernonia anthelmintica*, a plant traditionally used in herbal medicine, and identified its flavonoid component, kaempferol, as a potent antioxidant. Kaempferol was shown to enhance antioxidant enzyme activity and suppress ROS generation in keratinocytes, demonstrating its potential therapeutic application in oxidative stress-related skin disorders. These findings highlight the synergistic interactions between natural bioactive compounds and key biochemical pathways, offering valuable insights into the development of antioxidant-based therapeutic strategies.

## Pediatric considerations: the role of oxidative stress in childhood disorders

Oxidative stress and inflammation pose significant risks in pediatric populations, where they contribute to long-term complications and disease progression. Lupu et al. investigated the impact of oxidative stress in pediatric diabetes, emphasizing its role in systemic decline, including damage to pancreatic islets, retinas, kidneys, and the cardiovascular system. Their findings underscored the critical role of antioxidants, such as vitamins C and E, polyphenols, and essential minerals, in mitigating oxidative damage and preserving metabolic function. Given the heightened vulnerability of children to oxidative stress-related disorders, early prevention strategies must be prioritized. Recognizing the profound influence of nutrition and antioxidant therapy in shaping long-term metabolic and cardiovascular health is essential for developing effective pediatric interventions.

## Lifestyle and population health: insights from epidemiology

Large-scale population studies offer valuable insights into the intricate relationship between oxidative stress, lifestyle factors, and metabolic disorders. The study by Wen et al. on CKD demonstrated that a higher OBS, indicative of a diet and lifestyle rich in antioxidants, is inversely associated with CKD prevalence. These findings reinforce the pivotal role of lifestyle interventions as a foundation for disease prevention. Similarly, Yang et al. reported that a higher OBS correlates with lower serum uric acid levels, further highlighting the systemic benefits of maintaining oxidative balance.

These findings support the implementation of public health initiatives that encourage antioxidant-rich dietary patterns—including fruits, vegetables, and whole grains—while minimizing pro-oxidant exposures from processed foods and environmental toxins. Such strategies not only mitigate individual disease risk but also alleviate the broader healthcare burden associated with metabolic disorders.

## Challenges and future directions

Despite significant advancements, several challenges persist in translating these findings into clinical practice. Biomarkers such as CLDN2 and assessment tools like the OBS require further validation across diverse populations to ensure reliability and seamless integration into healthcare systems. Similarly, therapeutic agents, including DPDS and kaempferol, necessitate additional clinical trials to establish their long-term efficacy and safety profiles.

Addressing these challenges requires interdisciplinary collaboration between clinical researchers, biochemists, and healthcare professionals. A deeper understanding of the bidirectional relationship between gut health, oxidative stress, and systemic inflammation, as highlighted by Wang et al., could pave the way for novel therapeutic interventions. Strategies leveraging probiotics, prebiotics, and targeted dietary modifications may offer promising avenues to modulate oxidative stress and inflammation, ultimately improving metabolic health outcomes.

## Conclusion

The studies discussed in this editorial underscore the pivotal role of oxidative stress and inflammation in the pathogenesis of metabolic disorders. Collectively, they emphasize the significance of early detection through biomarkers, the therapeutic potential of targeting oxidative pathways, and the critical impact of lifestyle modifications in disease prevention. From pediatric diabetes to CKD and hyperuricemia, oxidative stress functions both as a biomarker and a key mediator of disease progression. Looking ahead, the integration of molecular insights with population health strategies will be essential in addressing the growing burden of metabolic disorders. Bridging basic research with clinical practice can facilitate the development of targeted interventions that not only treat but also prevent these conditions. The future of metabolic disorder management lies in personalized, interdisciplinary approaches that address oxidative stress and inflammation at their roots, paving the way for improved preventive and therapeutic outcomes.

